# The clinical characteristics, mechanism and management of immune checkpoint inhibitor-related arthritis

**DOI:** 10.3389/fimmu.2025.1611692

**Published:** 2026-01-14

**Authors:** Jian Gao, Jinlin Miao, Zhinan Chen, Ping Zhu

**Affiliations:** 1 National Center for International Research of Bio-targeting Theranostics, Guangxi Key Laboratory of Bio-targeting Theranostics, Collaborative Innovation Center for Targeting Tumor Diagnosis and Therapy, Guangxi Talent Highland of Bio-targeting Theranostics, Guangxi Medical University, Nanning, China; 2 Department of Clinical Immunology of Xijing Hospital and Department of Cell Biology of National Translational Science Center for Molecular Medicine, Fourth Military Medical University, Xi’an, China

**Keywords:** arthritis, immune checkpoint inhibitors, immunotherapy, irAEs, tumor

## Abstract

**Background:**

Immune-related adverse events, notably arthritis (irAE-arthritis), frequently occur in patients receiving immune checkpoint inhibitors. Arthritis severity varies from mild to severe, adversely impacting quality of life. Despite reports in clinical trials and real-world studies, the pathophysiology and optimal management of irAE-associated arthritis (irAE-arthritis) are still unclear.

**Methods:**

From the inception to September 25, 2025, a search was conducted on PubMed, EMBASE, and MEDLINE for case reports/series on irAE-arthritis.

**Findings:**

The most common rheumatic irAEs were arthritis. Various rheumatic syndromes have been reported, such as arthralgia, mono-/oligo-/polyarthritis, reactive and psoriatic arthritis, RS3PE, tenosynovitis. The onset of irAE-arthritis is attributed to T cell dysregulation, B cell activation with autoantibody production, cytokine - mediated inflammation, and impaired immune tolerance due to Treg dysfunction. NSAIDs, intra-articular/systemic corticosteroids, csDMARDs, and biologics play key roles in irAE-arthritis management, and JAK inhibitors may emerge as a significant therapeutic strategy in the future.

**Conclusion:**

Given the increasing use of immunotherapy in oncology and other fields, developing a comprehensive understanding of irAE-arthritis is crucial. This review aims to provide an in-depth overview of current knowledge on irAE-arthritis, including its epidemiology, clinical presentation, underlying mechanisms, and management approaches.

## 1 Introduction

Immunotherapy has ushered in a transformative era in cancer treatment. A prominent category of cancer immunotherapy is immune checkpoint inhibitors (ICIs), which disrupt immune regulatory interactions to enhance T cell activation and elicit anti-tumor immune responses (1, 2). ICIs are employed across an ever-growing spectrum of tumor types. However, their use is also associated with adverse events known as immune-related adverse events (irAEs), which represent inflammatory responses resulting from the non-specific activation of the immune system. irAEs can affect various tissues and manifest with a variety of clinical symptoms (3, 4). Consequently, irAEs exhibiting phenotypes akin to rheumatoid arthritis (RA), spondyloarthritis, polymyalgia rheumatica (PMR), and other rheumatic disorders (referred to as rheumatic irAEs) have been described (5). Given that over 40% of oncology patients qualify for ICI treatment, the potential number of individuals experiencing arthritis is substantial (6).

The risk factors for irAE-arthritis remain largely undefined in current studies. Notably, different classes of ICIs have been associated with distinct profiles of irAEs. For instance, certain irAEs such as rash, colitis, and hypophysitis appear to be more prevalent with cytotoxic T lymphocyte antigen 4 (CTLA-4) blockade, whereas pneumonitis, arthritis, and hypothyroidism are more commonly observed with programmed cell death receptor 1 (PD-1) blockade (7). Additionally, rheumatic irAEs, particularly inflammatory arthritis, have been documented to persist even after the cessation of ICI therapy (8). This persistence is notably associated with several factors, including the use of combination ICI regimens, prolonged durations of ICI treatment, and the presence of multiple irAEs (8).

The coexistence of cancer and the objective of eliciting an immune response against tumors presents complexity and challenges in managing irAEs. Clinicians must carefully balance the need to reduce inflammation in affected organs with the imperative to preserve the anti-tumor efficacy of ICIs. On a more optimistic note, certain irAEs, such as arthritis, have been identified as a positive prognostic indicator for tumor response across various cancer types (9). Furthermore, arthritis is a relatively common side effect of ICIs. To develop optimal management strategies that do not compromise anti-tumor immunity, it is crucial to further clarify the clinical presentations of irAE-arthritis and to explore their underlying mechanisms. A deeper understanding of these aspects could facilitate more tailored treatment approaches, allowing clinicians to effectively navigate the complexities of concurrent cancer treatment and severe immunological responses, ultimately improving outcomes for patients undergoing ICI therapy.

### 1.1 Immune checkpoint

**Figure 1:**
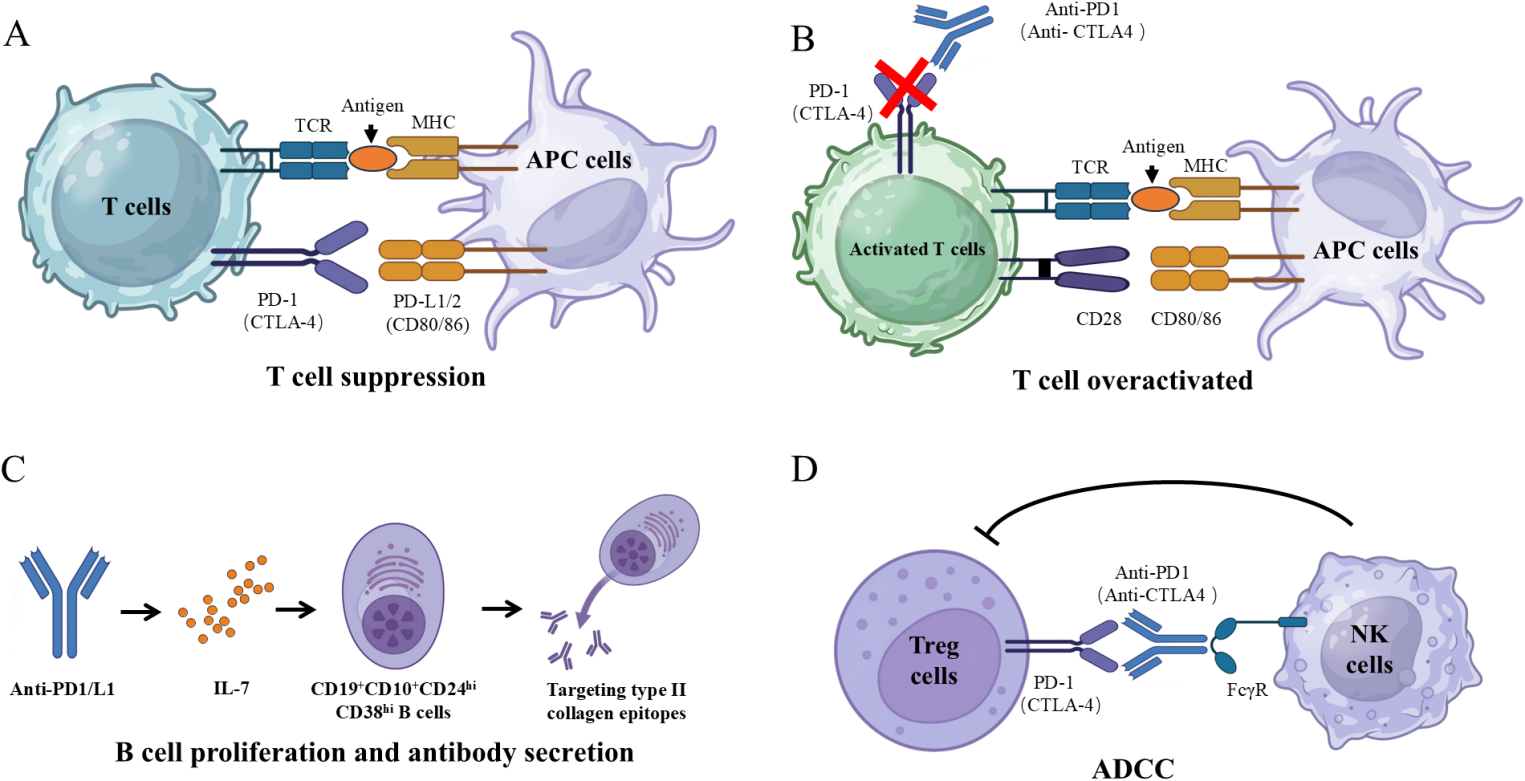
Mechanistic pathways of irAE-arthritis. **(A)** The T cell receptor (TCR) on T cells recognizes antigen-MHC complexes on APCs, triggering T-cell activation. The binding of PD-1 and CTLA-4 to their respective ligands on APC inhibits T-cell signaling, leading to decreased activity and increased apoptosis. **(B)** The interaction of CD80/86 with CD28 enhances T-cell activation, whereas CTLA-4 competes for these ligands, inhibiting activation. Activated T cells that express PD-1 bind to PD-L1, further inhibiting signaling pathways. The blockade of PD-1 and CTLA-4 results in T-cell hyperactivation. **(C)** The Fab region of an anti-PD-1/CTLA-4 antibodies targets surface epitopes on regulatory T cells (Tregs), specifically CTLA-4 and PD-1. The Fc region of these antibodies interacts with Fc receptors on immune effector cells, particularly natural killer (NK) cells, which subsequently disrupt the target cell membrane and induce cell lysis. **(D)** Inhibition of the PD-1/PD-L1 pathway enhances interleukin-7 (IL-7) secretion, which promotes B-cell differentiation and proliferation, ultimately increasing the production of autoantibodies that target type II collagen epitopes.

Immune checkpoints, highly expressed on activated T and B cells, are vital for immune regulation and balance. CTLA-4 and PD-1 are the most studied, playing key roles in negatively regulating T cell activation (10). CTLA-4 outcompetes CD28 by binding CD80/CD86 with higher affinity, blocking T cell activation. Tregs’ high CTLA-4 expression is crucial for tumor immune evasion (11). Conversely, the interaction between PD-1 and its ligands, PD-L1 and PD-L2, serves as a negative regulator of T cell function, maintaining a delicate balance between T cell activation, tolerance and immune-mediated tissue damage (12, 13). PD-1 is highly expressed on Tregs, promoting their proliferation via ligands and suppressing anti-tumor immunity due to tumor-infiltrating Tregs. Blocking PD-1 enhances anti-tumor immunity by reducing Treg inhibition (14). These immune checkpoints are essential for maintaining immune homeostasis and ensuring the tolerance of normal tissues, thereby protecting organs from unnecessary damage while allowing the immune system to effectively eliminate pathogens (15) (**Figure 1A**). Other immune checkpoints, such as T cell immunoglobulin-3 (TIM3) (16), lymphocyte activation gene 3 protein (LAG3) and T cell immunoreceptor with Ig and ITIM domains (TIGIT) also play important roles in immunity regulation (17). Since the FDA’s 2011 approval of ipilimumab (the first CTLA-4-targeting ICI), cancer therapy, particularly for advanced melanoma, has undergone a revolutionary shift (18–20). Subsequently, pembrolizumab and nivolumab, targeting PD-1, received FDA approval from 2014 to 2015, for the treatment of advanced melanoma, mismatch repair defects (dMMR) and microsatellite instability-high (MSI-H) cancers and non-small cell lung cancer (20, 21). The approval of ipilimumab and nivolumab combination immunotherapy for advanced melanoma marked a milestone, yielding significant therapeutic benefits (20). Since May 2016, the FDA has OK’d three PD-L1 drugs—atezolizumab, durvalumab, and avelumab—for treating non-small cell lung and urothelial cancers (22–24). Overall, ICIs have revolutionized the field of oncology, significantly enhancing the long-term survival rates for patients with various types of cancer.

### 1.2 Immune-related adverse events

ICIs have revolutionized patient care in oncology, leading to a distinctive spectrum of organ-specific inflammatory toxicities known as irAEs (4). These toxicities are notably common, with approximately 50% of patients treated with ICIs experiencing some form of irAEs (25). A systematic review of ICI-irAEs from randomized controlled trials (RCTs) reported that 14% of patients treated with PD-1/PD-L1 inhibitors, 34% of patients receiving CTLA-4 inhibitors, 55% of patients undergoing combination ICI therapy, and 46% of patients receiving immunotherapy combined with chemotherapy experienced grade ≥ 3 irAEs (26). Interestingly, the occurrence of irAEs is usually associated with a more favorable response to tumor treatment (27). Among these adverse events, rheumatologic immune-related adverse events (rheumatic irAEs) represent a specific category of side effects induced by immunotherapies, including ICI-related arthralgia and arthritis, with reported frequencies ranging from 1% to 43% (28), highlighting their prevalence among rheumatic irAEs.

## 2 Epidemiology and clinical manifestation of immune-related arthritis

### 2.1 Prevalence

IrAEs occur more frequently in patients receiving both anti-PD-1 and anti-CTLA-4 inhibitors compared to those undergoing monotherapy (29). Interestingly, irAE-arthritis, reported to affect approximately 1-7% of patients (**Table 1**), did not exhibit a female predominance in these studies (30, 31). These events may emerge at any point during therapy, with onset occurring from 2 weeks to over a year after the initiation of ICI therapy (8, 32, 33). Notably, anti-PD-1/L1 therapies have been associated with a higher incidence of rheumatic irAEs compared to anti-CTLA-4 monotherapy (13, 34, 35). IrAE-arthritis can arise from either CTLA-4 or PD-1 inhibitor monotherapy or from combination therapy involving both agents (33). The incidence rates of arthralgia vary among different treatments: 9% to 12% in patients receiving pembrolizumab, 6% to 8% in those treated with nivolumab, 5% for ipilimumab, and 11% for patients undergoing combination therapy with nivolumab and ipilimumab (36). Interestingly, irAE-arthritis is predominantly polyarticular, affecting approximately 65% of patients, while about 35% experience oligoarticular involvement (37) (**Table 1**). Additionally, the prevalence of irAE-arthritis does not appear to correlate significantly with age and gender (38).

**Table 1:** IrAE-arthritis clinical features.

Characteristic	Value	Reference
Prevalence incidence		
Any immune checkpoint inhibitors	7%	(30)
ipilimumab	5%	(36)
nivolumab	6-8%	(36)
pembrolizumab	9-12%	(36)
nivolumab plus ipilimumab	11%	(36)
Polyarthralgia	65%	(37)
Oligo-arthralgia	35%	(37)
Arthropathy symptoms onset time	Typical onset time	
IrAE-arthritis	2–24 months	(31)
Rheumatoid arthritis (RA)	1–4 days	(50)
	0.1–2 months	(51)
	7days	(52)
Psoriatic arthritis (PsA)	4–31 days	(50)
	6–17 weeks	(53)
	2 weeks	(54)
	2 weeks	(55)
	5mounths	(56)
	5mounths	(57)
Remitting Seronegative Symmetric Polyarthritis with Pitting Edema (RS3PE)	4–126 weeks	(58)
Polyarthritis	2–27 days	(50)

### 2.2 Clinical subtypes (polyarthritis, oligoarthritis, PsA, RS3PE)

IrAE-arthritis is characterized by joint stiffness and swelling (39). Additionally, a variety of other rheumatic syndromes have been reported, including arthralgia; mono-arthritis, oligoarthritis or polyarthritis; reactive arthritis; psoriatic arthritis (PsA); remitting seronegative symmetrical synovitis with pitting oedema (RS3PE); tenosynovitis; enthesitis; non-inflammatory musculoskeletal conditions; and osteoarthritis (39, 40). One earlier study described 9 patients who developed inflammatory arthritis as a result of ICIs, with some of presenting additional symptoms such as urethritis and conjunctivitis, which are consistent with a diagnosis of reactive arthritis (41). Unusual manifestations reported in case studies include inflammatory tenosynovitis affecting the hands and/or shoulders, as well as enthesitis and swan-neck deformities indicative of Jaccoud’s arthropathy (42–44). Moreover, some patients with pre-existing osteoarthropathy experienced aggravation of joint inflammation, indicating that inflammatory responses can be triggered in individuals with degenerative joint diseases following ICI treatment (45).

In general, imaging modalities such as MRI and musculoskeletal ultrasonography play a crucial role in identifying erosive disease, tenosynovitis, doppler-positive synovitis, and joint effusions in patients with inflammatory arthritis (41, 46). Medical imaging, including PET-MRI, has been effective in diagnosing inflammatory arthritis, demonstrating fluorodeoxyglucose uptake in the synovial tissue of multiple peripheral bilateral joints, and these manifestations were observed between 2 to 26 months following ICI therapy (47). According to Radiographic grading system for arthritis (**Table 2**), point-of-care musculoskeletal ultrasound (MSKUS) is a valuable tool for evaluating potential irAE-arthritis (48). The most commonly identified MSKUS features include grade 2 or higher synovial thickening (80%), hyperemia measured by color power Doppler signal (70%), and tenosynovitis (60%) (49).

**Table 2:** Radiographic grading system for arthritis.

Grade	Description
0	Intact bony outlines and normal joint space
1	Erosion < 1 mm in diameter or joint space narrowing
2	One or several small erosions, diameter > 1 mm
3	Marked erosions
4	Severe erosions, where there is usually no joint space left and the original bony outlines are partly preserved
5	Mutilating changes, where the original bony outlines have been destroyed

### 2.3 Typical onset time

Inflammatory polyarthritis is a frequently reported manifestation in patients undergoing ICI therapy. Clinical investigations showed that the majority of irAE-arthritis patients have received anti-PD-1/PD-L1 agents. Among these patients, a study showed that symptoms of arthropathy typically present with a time to onset of about 2–24 months following the initiation of ICI treatment (31). Additionally, some patients developed RA after ICI treatment, with a time to onset ranging from 1days to 2 months (50–52). PsA has been documented in six patients who were undergoing Immune Checkpoint Inhibitor (ICI) therapy, with the condition manifesting approximately 0.5 to 5 months after treatment initiation (50, 53–57). RS3PE was observed in 11 patients, with a median time to onset of 26 weeks (range: 4–126 weeks) from the start of ICI therapy (58). Similarly, the onset of polyarthritis was reported with a median time of 8 days (range: 2–27 days) (50) (**Table 1**).

### 2.4 Associated ICIs

Combining anti-CTLA-4 and PD-1/PD-L1 therapies can worsen arthritis. Two prior studies linked CTLA-4 (ipilimumab) and PD-1 inhibitors (nivolumab, pembrolizumab) to exacerbated RA and psoriatic arthritis (59, 60). The observed clinical features primarily included exacerbations of pre-existing disease, with no new manifestations of autoimmune disease reported (61).

Overall, anti-PD1 is more likely to induce the irAE-arthritis, and combination therapy significantly increases both the incidence and severity of these adverse events. Although arthritis symptoms often arise within the first few months of initiating ICI therapy, there have been instances of delayed onset arthritis that can persist even after the discontinuation of ICI treatment. Moreover, among patients with irAE-arthritis, approximately half also experienced non-rheumatic immune-related adverse events (62).

## 3 Mechanisms of irAE-arthritis

The underlying pathogenesis of irAE-arthritis remains largely unclear. Early reports suggested that ICI-associated inflammatory polyarthritis predominantly occurred in seronegative patients (5). However, more recent findings indicate that individuals who are positive for anti-citrullinated protein antibody (ACPA) prior to ICI treatment have an increased risk of experiencing sudden onset arthritis, particularly with anti-PD-1 therapies (51). In cases of irAE-arthritis, while rheumatoid factor (RF) or anti–cyclic citrullinated peptide (CCP) antibodies are often present, their prevalence is notably lower compared to that in traditional RA without ICI involvement (63, 64). Notably, the presence of pre-existing RF or CCP antibodies, without clinical evidence of arthritis at the initiation of ICI therapy, has been correlated with an increased risk of developing irAE-arthritis (65). Additionally, a study has provided compelling evidence suggesting that defective PD-1 inhibitory signaling may significantly contribute to RA pathogenesis, indicating a downregulation of the PD-1 pathway during the progression of RA (66).

### 3.1 T cell dysregulation

Immune checkpoint molecules are predominantly expressed on the surface of T cells, where they play a critical role in dampening the ongoing T cell response (67). ICIs primarily target these T cells, counteracting the function of these molecules by disrupting their interactions with ligands. This interference impairs the effective modulation of immune cell activation and proliferation, thereby destabilizing the balance of the immune system and making it more susceptible to hyperactivation (68, 69). Observations indicate that patients who progress to irAEs following anti-CTLA-4 therapy exhibit a tendency for the expansion of autoreactive CD4^+^ T cells, along with the proliferation of specific T cell clonal subsets, suggesting that anti-CTLA-4 treatment can elicit the differentiation and diversification of the T cell repertoire (70). When ICIs are administered systemically, they can activate autoreactive T cell clones that target autoantigens (**Figure 1B**). The majority of these activated T cells lack specificity, which can lead to systemic multi-organ toxicity and potentially life-threatening conditions (71).

#### 3.1.1 CD4^+^ T cells

In a recent study, Bukhari et al. employed single-cell RNA sequencing (scRNA-seq) and cellular indexing of transcriptomes and epitopes (CITE-seq) to analyze PBMCs from a heterogeneous cohort of 18 lung cancer patients, 4 prostate cancer patients, and 2 head and neck cancer patients treating with various ICI regimens (anti-PD-1, anti-PD-L1, or combination ICIs) (72). Gene set analysis revealed increased expression of cytotoxic and pro-inflammatory genes in CD4^+^ T effector and memory subsets in patients who developed irAE compared to those without. The authors then identified distinct CD4^+^ T cell clusters and attempted to correlate them with organ-specific irAEs. Notably, a cluster of CD4^+^ T helper 1/2 (Th1/2) cells was significantly elevated at baseline in patients who later developed irAE-arthritis, while no differences were observed in patients with pneumonitis or thyroiditis. Meanwhile, this specific CD4^+^ Th1/2 population exhibited low levels of CCR7 and TCF7, suggesting an effector memory phenotype (72). Further gene expression profiling of the Th1/2 cells revealed substantial upregulation of inflammatory genes. Gene ontology and network analysis showed substantial overlap with genes related to activated CD4^+^ T cell responses and autoimmune conditions, including inflammatory arthritis. Additional analysis revealed that pneumonitis patients had a specific Th2 cell state enriched in peripheral blood at baseline, while thyroiditis patients exhibited elevated baseline levels of Th17 cells (72). Moreover, Th17 cells were found to be significantly enriched in synovial fluid (SF) from patients with irAE-arthritis following combined anti-CTLA-4 and PD-1 therapy. This treatment preferentially enhanced Th17 and transient Th1/Th17 cell signatures. In addition, Th17 and cytotoxic T cell-17 (Tc17) cells may contribute to steroid resistance in irAE-arthritis, particularly in cases linked to combined ICI therapies (63).

#### 3.1.2 CD8^+^ T cells

Compared to CD8^+^ T cells from patients receiving ICIs without irAEs, peripheral CD8^+^ T cells from those with irAEs showed significantly lower frequencies and expression levels of cell-surface molecules associated with activation, effector-functions, homing, exhaustion and apoptosis. Additionally, there was a diminished release of cytotoxic and proinflammatory immune mediators (73). Meanwhile, these alterations were accompanied by an increased glycolytic rate and enhanced ATP production within the CD8^+^ T cells. Gene expression analysis of pre-ICI-treated CD8^+^ T cells revealed several differentially expressed transcripts in patients who subsequently developed irAE-arthritis (73), indicating an intrinsic predisposition that may influence subsequent development of these adverse events. A study that comprehensively analyzed peripheral blood and/or synovial fluid samples from 20 patients with irAE-arthritis uncovered a prominent Th1-CD8^+^ T cell axis present in both blood and inflamed joints (63). The study found that CX3CR1^hi^ CD8^+^ T cells in blood and CXCR3^hi^ CD8^+^ T cells in synovial fluid represented the most clonally expanded T cells, sharing similar T cell receptor (TCR) repertoires. The migration of blood CX3CR1^hi^ CD8^+^ T cells into the joints may be facilitated by chemokines such as CXCL9, CXCL10, CXCL11, and CXCL16, which are expressed by myeloid cells within the inflamed environment (63). Furthermore, combination therapy with anti-PD-1 and anti-CTLA-4 preferentially expanded autoreactive effective T cells (74, 75). Collectively, these data suggest that effective CD8^+^ T cells contribute to irAE-arthritis. In summary, ICIs suppress the regulation of immune checkpoints, resulting in the progressive accumulation of activated T cells that ultimately trigger inflammation (**Figure 1B**).

### 3.2 B cell involvement and autoantibodies

Recent study showed that IL-7 is implicated in multiple stages of B cell progenitor development, including commitment, survival, differentiation, and proliferation (76). Furthermore, the interaction between PD-1 and PD-L1 can induce apoptosis in B cells (77). A particular genetic variant, rs16906115, has been shown to elevate IL-7 expression in the B cells of patients with irAEs who carry the risk allele. This increased IL-7 expression is independently associated with a higher likelihood of experiencing irAEs, alterations in antibody production, and a greater frequency of mutations in B cell receptors (78). Consequently, PD-1/L1 inhibitors can increase the IL-7 secretion to promote B cell proliferation and differentiation (77, 79) (**Figure 1C**). Recent research also indicates that patients with irAE-arthritis have higher proportions of CD19^+^ B cells compared to those without irAEs (80). Furthermore, individuals with irAE-arthritis exhibit an increase in both the proportion and absolute numbers of transitional CD19^+^CD10^+^CD24^hi^CD38^hi^ B cells compared to non-irAE patients. Specifically, these transitional B cells increase prior to the onset of overt irAE-arthritis symptoms and decrease during the transition between the active and quiescent disease stages. Additionally, autoantibodies targeting type II collagen epitopes have been identified in up to 43% of irAE-arthritis patients, whereas no such autoantibodies were observed in non-irAE patients (80). Therefore, the increased proportion of B cells results in the secretion of autoantibodies, ultimately leading to the development of irAE-arthritis (**Figure 1D**).

### 3.3 Cytokine-driven inflammation

#### 3.3.1 TNF-α

TNF-α is a critical mediator in various systemic inflammatory diseases. Anti-TNF-α therapies have proven effective in managing inflammation associated with several autoimmune diseases, including RA (81) and ankylosing spondylitis (AS) (82). Notably, a study reported that TNF-α levels were significantly elevated following 3 months of anti-PD-1 therapy in NSCLC patients (83). Furthermore, our previous study indicated a significantly higher frequency of TNF-α secreting T cells in a humanized mouse model of ICI-related arthritis and pneumonitis (74). Additionally, patients exhibiting elevated TNF-α levels showed an increased likelihood of experiencing severe adverse events (SAEs) (≥ grade 3), whereas no SAEs were observed in patients with normal TNF-α levels (84). Gene expression analysis further revealed that inflammatory molecules, including TNF-α, were significantly upregulated across all T cell subclusters from irAE-arthritis patients (63).

#### 3.3.2 IFN-γ

IFN-γ is a multipotent cytokine with pro-inflammatory and immunomodulatory properties, contributing to the development of arthritis (85). IFN-γ facilitates the differentiation of lymphocytes and macrophages through the JAK-STAT signaling pathway, integral to the pathogenesis of arthritis (86). Additionally, IFN-γ is essential for PD-L1 and PD-L2 expression, serving as a biomarker for responses to ICI (87). Anti-PD-L1 treatment has been linked to elevated circulating IFN-γ levels (88, 89). Furthermore, subcluster analyses revealed IFN-γ-producing Th1/Tc1 cells may be pivotal in the pathogenesis of irAE-arthritis (63). Intracellular staining of peripheral blood and synovial fluid samples from irAE-arthritis patients reveals a significant presence of IFN-γ-secreting T cells (63). These findings highlight the critical role of IFN-γ-producing T cells in the inflammatory processes underlying irAE-arthritis.

#### 3.3.3 IL-6

Clinical research has shown that patients with elevated IL-6 levels following initial anti-PD-1 treatment have an increased incidence of irAEs and SAEs (84). IL-6 is produced by various cell types, including macrophages, T cells, B cells, and synovial fibroblasts (90). It serves as a pivotal cytokine within the hierarchical cytokine network implicated in the pathogenesis and progression of RA. The multifaceted roles of IL-6 facilitate the inflammatory cascade and joint destruction characteristic of this autoimmune disease (91, 92). Additionally, IL-6 can activate the JAK-STAT pathway, further exacerbating arthritis development (86). Its diverse functions encompass B cell proliferation and antibody production, hematopoiesis, and T cell differentiation (91, 92). Consequently, elevated IL-6 levels following anti-PD-1 treatment may lead to the development of irAE-arthritis.

#### 3.3.4 Other cytokines and chemokines

Further evidence of generalized immune activation during ICI therapy underscores the increased production of inflammatory cytokines (93). Previous studies have demonstrated a significant correlation between baseline circulating IL-17 levels and the incidence of high-grade irAEs (94). Additionally, elevated circulating levels of IL-1 have been identified as a predictive marker for irAE-arthritis in melanoma patients (95). Moreover, the CXCL9/10/11 chemokine system has been implicated in the pathogenesis of irAE-arthritis (63).

### 3.4 Loss of immune tolerance (Tregs)

It is widely recognized that CD4^+^CD25^+^Foxp3^+^ Tregs play a crucial role in maintaining immune homeostasis, and their interaction with co-suppressor molecules is critical for attenuating the activation of CD4^+^ and CD8^+^ T cell responses (96). Recent research has illuminated that ICIs lead to a reducing frequency of CD4^+^CD25^+^Foxp3^+^ Tregs (75). This reduction is partly attributed to the constitutive expression of CTLA-4, rendering antigen-specific Tregs susceptible to macrophage-mediated antibody-dependent cell-mediated cytotoxicity (ADCC), ultimately resulting in Treg depletion (75, 97), which may induce arthritis (**Figure 1C**). Intriguingly, transcriptomic analyses of Tregs suggested that these cells are markedly enriched in synovial fluid of patients with irAE-arthritis and exhibit effective Treg (eTreg) phenotypes characterized by enhanced suppressive functions compared to those their counterparts in the peripheral blood (63).

Moreover, PD-1 is mainly expressed in specific Treg subsets. The PD-1/PD-L1 pathway potentiates anti-tumor immunity by inhibiting iTregs production and Foxp3 expression, compromising Treg function and potentially breaking self-tolerance (96, 98). Additionally, a recent study highlighted significant inflammatory transcriptional reprogramming of Tregs in the periphery of cancer patients who developed irAEs following anti-PD-1 immunotherapy. Notably, transcripts such as IFNG, STAT1, RORC and STAT3 were found to be highly enriched in these Tregs (99). This finding underscores the reprogramming of peripheral Tregs toward an inflammatory signature upon ICI immunotherapy in individuals with irAEs. Further, Tregs from patients with irAEs demonstrated metabolic reprogramming (99), which aligns with the dysfunction of Tregs observed in various autoimmune diseases (100).

In summary, ICI therapy may enhance the secretion of inflammatory cytokines, thereby promoting the occurrence and development of irAE-arthritis.

## 4 Medications for irAE-arthritis

**Figure 2:**
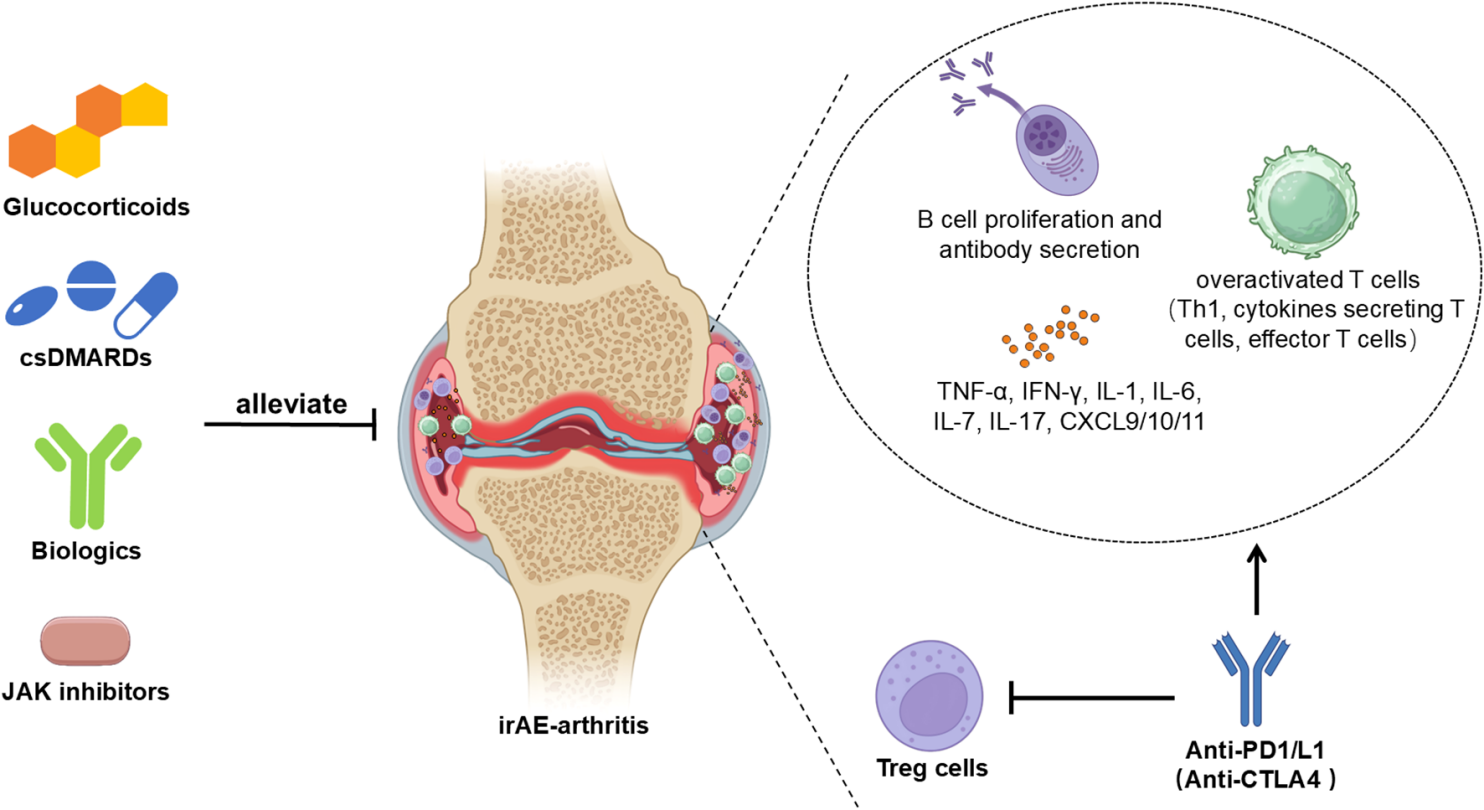
Overview of therapeutic strategies. The pathogenesis of irAE-arthritis is characterized by the accumulation of overactivated T cells and inflammatory cytokines, coupled with B-cell proliferation and increased autoantibody secretion. Current therapeutic strategies encompass glucocorticoid therapy, conventional synthetic disease-modifying antirheumatic drugs (csDMARDs), biological agents, and Janus kinase (JAK) inhibitors.

The management of rheumatic irAEs presents a complex challenge for clinicians, including both rheumatologists and oncologists (**Figure 2**). Treatment decisions must carefully weigh the risks and benefits, tailored to individual circumstances, considering oncologic prognosis, response to ICIs, and the severity of the irAEs (101). Recent guidelines from the European League against Rheumatism (EULAR) recommend that the initial management of mild-to-moderate rheumatic irAEs should focus on symptomatic treatment, including non-steroidal anti-inflammatory drugs and/or analgesics (39).

### 4.1 First-line: NSAIDs and intra-articular corticosteroids

For patients with milder forms of arthritis, nonsteroidal anti-inflammatory drugs (NSAIDs) are typically recommended as the first-line treatment. In cases of monoarthritis or oligoarthritis, intra-articular corticosteroids (CSs) can provide more targeted management (41).

### 4.2 Second-line: systemic corticosteroids

Many patients may require systemic CSs, typically initiated at moderate doses of 10–20 mg of prednisone. Some patients may necessitate long-term, low- to moderate-dose CSs to facilitate continued treatment with ICIs in oncology (39).Certain rheumatic irAEs may require higher doses of CSs, and case series have shown that arthritis, which typically responds to low–medium doses, might require higher doses when treating irAEs (41). Additionally, exogenous CSs at clinically relevant concentrations exert immunosuppressive effects by impairing the ability of dendritic cells to present tumor antigens, as well as inhibiting T cell activation and anti-tumor activity (102). However, a systematic literature review indicated that the concomitant administration of CSs and ICIs may not necessarily lead to poorer clinical outcomes (103).

Studies in mouse models have shown that both endogenous and exogenous glucocorticoids can inhibit anti-cancer immune responses (104). One particular study assessed the influence of clinically relevant doses of dexamethasone and an anti-TNF monoclonal antibody *in vitro*, revealing that even low doses of CSs markedly impaired the anti-tumor activity of tumor-infiltrating lymphocytes, although this activity was restored upon withdrawal of steroids (105). Additionally, recent studies have revealed that CSs significantly abrogate the anti-tumor efficacy of ICIs at doses of ≥10 mg of daily prednisone or its equivalent (106, 107). Consequently, the therapeutic use of glucocorticoids for managing irAE-arthritis requires careful consideration and caution.

### 4.3 Third-line: csDMARDs (e.g., methotrexate, hydroxychloroquine)

Early referral to a rheumatologist is recommended for patients exhibiting grade ≥2 symptoms prior to initiating corticosteroid therapy, particularly if there is an insufficient response to acceptable doses of CSs, or if a corticosteroid-sparing regimen is necessary. For these patients, conventional synthetic disease-modifying antirheumatic drugs (csDMARDs) should be considered, including methotrexate, hydroxychloroquine, or sulfasalazine (39). It is important to note that many patients with irAE-arthritis experience a milder disease course with clinical manifestations that deviate from typical inflammatory chronic arthritis and systemic rheumatic diseases. Consequently, the need for CSs or DMARDs may be diminished (108).

Recent clinical reports indicate that patients with irAEs who gradually reduced their does of CSs can obtain long-term remission following methotrexate treatment, and follow-up studies found that methotrexate exhibits favorable efficacy and safety, especially in cases of multiple irAE-arthritis (109). Additionally, a case report described that hydroxychloroquine was used in patients with inflammatory arthritis who exhibited minimal improvement in arthralgias after treatment with high-dose steroids. Nevertheless, during this treatment period, imaging evaluations revealed significant progression of melanoma, characterized by new bilateral lung metastases and an enlarging left inguinal adenopathy (110). Furthermore, other agents such as sulfasalazine, leflunomide, azathioprine, and apremilast have also proven to be highly effective in the management of irAE-arthritis (111).

### 4.4 Fourth-line: biologics (e.g., anti-TNF, anti-IL-6, anti-IL-17, anti-IL-1)

Mild irAE-arthritis is initially treated with NSAIDs. If irAE-arthritis progresses to moderate or severe stages, CSs are typically initiated and ICI(s) may need to be withheld. In cases where irAE-arthritis does not adequately respond to CSs or the steroids cannot be tapered off (termed steroid resistance), steroid-sparing DMARDs are administered (112).

For severe irAEs or cases exhibiting inadequate responses to csDMARDs, biologic DMARDs (bDMARDs) may be considered. Over the past two decades, the development of selective immune-targeted therapeutics based on pathogenesis-driven principles has significantly expanded the therapeutic options available for various rheumatic diseases. Numerous monoclonal antibodies targeting key pro-inflammatory cytokines and immune cells have been proven effective against chronic rheumatic conditions while maintaining an acceptable safety profile (113). Notably, for arthritis cases, TNF and IL-6 blockade have been successfully employed. These two target cytokines play central roles in the immunopathogenesis of irAE-arthritis.

#### 4.4.1 Anti-TNF-α

TNF-α, pivotal in physiological and pathological processes by driving inflammatory disorders, has its autoimmune link to irAEs highlighted by TNF-α blockade’s ability to relieve irAE symptoms in mice (114, 115). TNF inhibition has also proven effective in treating severe colitis and arthritis (116). For arthritis, TNF inhibitors are recommended following the failure of first-line treatments, including glucocorticoid and csDMARDs, particularly in severe cases (113). For example, a case report showed that inflammatory sternoclavicular joint arthritis induced by durvalumab demonstrates significant responsiveness to infliximab (117). Moreover, combining TNF inhibitors with CTLA4 and PD-1 immunotherapy has been shown to ameliorate colitis while enhancing anti-tumor efficacy (115). Our previous research indicated that anti-TNF-α could ameliorate ICI-related arthritis and pneumonitis in a humanized mouse model (74).

Several studies have explored the relationship between TNF-α levels and responses to ICI. Preclinical studies show improved outcomes when combining of TNF-α modulation with ICI therapy. For instance, compared to anti-PD-1 alone, the combination of anti-PD-1 and a TNF/TNFR1 gene defect or TNF blockade yielded a significantly increased survival rate (75%l vs. <20%) in melanoma and lung cancer mouse models (118). Clinically, Tanaka et al. showed that serum TNF-α levels decreased in 6 out of 9 patient with malignant melanoma who achieved complete remission (CR), partial remission (PR) or long-term stable disease (long SD), while TNF-α levels were elevated in 6 patients with progressive disease (PD) (119). Standard clinical doses of infliximab, a chimeric anti-TNF monoclonal antibody, had only a minimal effect on T cell activation and tumor cytotoxicity (105).This supports preclinical findings that TNF-α may induce resistance of immunotherapy (120). Interestingly, some case reports indicated that patients experiencing irAEs may respond positively to TNF-α inhibitors without adversely impacting the anti-tumor responses (8, 108, 113). This suggests a complex role for TNF-α in immunotherapy, where its inhibition might aid in managing irAEs while maintaining therapeutic efficacy against tumors.

#### 4.4.2 Anti-IL-6

Tocilizumab, targeting the IL-6 receptor, is approved and proven safe and effective for active RA treatment, alone or with methotrexate, in trials (121–123). In cases of severe inflammatory arthritis or inadequate response to csDMARDs, biologic agents such as IL-6R inhibitors are preferred over TNF-α inhibitors (39). In a study involving 87 patients who experienced irAEs following nivolumab treatment, clinical improvement was observed in 27 out of 34 patients treated with tocilizumab (124). A small case series also documented successful treatment of severe polyarthritis induced by ICIs with tocilizumab (125). Some irAE-arthritis patients who received CSs or other DMARDs as first-line therapies but had inadequate responses experienced a resolution or improvement of their irAEs to grade ≤1 after initiating anti-IL-6R antibody treatment (tocilizumab or sarilumab) (126). Moreover, the combined blockade of IL-6 and PD1/PD-L1 signaling has been shown to enhance tumor-specific Th1 responses and subsequent anti-tumor effects in tumor-bearing mouse models (127). Notably, treatment with IL-6R inhibitors has demonstrated therapeutical benefits without negatively impacting anti-tumor response (113, 124, 125). In addition to its efficacy in treating irAE-arthritis, tocilizumab has also proven effective as a secondary prophylactic therapy, effectively preventing symptom recurrence and extending the duration of ICI treatment following rechallenge (128). These findings suggest that targeting IL-6 signaling presents a viable strategy for managing irAE-arthritis in patients undergoing ICI therapy, while minimizing the risk of compromising anti-tumor immunity.

#### 4.4.3 Anti-IL-17

Secukinumab is an inhibitor of IL-17A that diminishes the IL-17A-driven contributions to autoimmune and inflammatory diseases (129). A case report showed the challenges of treating irAE-arthritis in the context of cancer immunotherapy. In this instance, an advanced melanoma patient developed inflammatory arthritis following treatment with ipilimumab and nivolumab (110). After initiating secukinumab, there was a gradual decline in inflammatory markers, and serial computed tomography (CT) imaging showed a significant reduction in size of his left inguinal adenopathy. After two months of ongoing monthly secukinumab treatment, the patient exhibited continued improvement in arthropathy symptoms, without significant progression of melanoma (110). Additionally, another study suggested that anti-IL-17A therapies during ICI treatment do not reduce the anti-tumor effects (130). Long-term results from a secukinumab trial revealed that over 80% of patients maintained their treatment for five years, underscoring its enduring therapeutic effectiveness (131, 132). Overall, these findings emphasize the necessity for carefully crafted management strategies that effectively address irAE-related arthritis while preserving the efficacy of cancer immunotherapy.

#### 4.4.4 Anti-IL-1

IL-1 is a crucial cytokine involved in the acute phase of inflammation (133). Preclinical studies have demonstrated that the IL-1β pathway significantly promotes tumor progression by activating tumor-associated macrophages and myeloid suppressive cells, as well as by upregulation of PD-L1 on tumor cells, which impairs effective immune responses (134). These findings underscore the potential therapeutic benefit of targeting the IL-1β pathway in conjunction with PD-L1 inhibition to enhanced anti-tumor effects. Anakinra, an IL-1 receptor antagonist, is currently approved for RA treatment and has also been used for managing various irAEs, including encephalitis, myocarditis, pneumonitis, and colitis (135). Notably, the blockade of this pathway has been associated with a reduced risk of cancer mortality (136). However, despite these promising insights, these agents are not incorporated into the current irAE treatment guidelines (137).

#### 4.4.5 Anti-CD20

Recent research showed that autoantibodies secreted by B cells may promote irAE-arthritis (80), leading to consideration of antagonistic B cell therapy. However, clinical studies specifically addressing irAE-arthritis remain relatively limited. Previous studies have demonstrated that rituximab can delay the onset of clinical RA (138). Additionally, another research reported that rituximab can ameliorate peripheral arthritis related to ICIs without significantly compromising anti-tumor efficacy (111).

### 4.5 Emerging: JAK inhibitors (limited data)

In recent years, a new category of DMARDs has emerged, known as targeted synthetic DMARDs (tsDMARDs), primarily consisting of the Janus Kinase (JAK) inhibitors. Clinical studies have reported that JAK inhibitors can be effective in treating ICI-related axial or peripheral arthritis (111). The activation of JAKs leads to the downstream activation of signal transducer and activator of transcription (STAT) proteins, which subsequently translocate to the nucleus to activate target genes (139). IL-6 is one of the cytokines that exerts its effects through the JAK/STAT pathway. Additionally, type I interferons transmits signals through JAK1 and JAK3, while type II interferon (IFN-γ) transmits signals through JAK2 (140). Considering the intimate relationship between the JAK/STAT pathway and inflammatory cytokines such as IL-6 and IFN (86), it is reasonable to hypothesis that JAK inhibition may aid in managing ICI-related adverse events. Indeed, preclinical studies have demonstrated that combining JAK inhibition with ICIs may help overcome resistance to ICI therapy, possibly through mitigating inflammation within the tumor microenvironment (141).

While various treatment options are currently available for irAE-arthritis, these therapies still have limitations, and further large-scale controlled studies are necessary to confirm their efficacy and safety. Additionally, there is an urgent need for the development of new therapeutic targets based on the underlying mechanisms of the irAE-arthritis.

## 5 Conclusions

A comprehensive initial assessment of irAE-arthritis should include joint count documentation and Radiographic grading system for arthritis to evaluate involvement extent, along with quantification of inflammatory markers and autoantibodies. Synovial fluid analysis, when feasible, can provide valuable insights. Imaging modalities are recommended to assess structural damage and inflammatory activity.

The pathogenesis of irAE-arthritis involves effector T cells and B cells, reflecting complex immune-inflammatory interactions during ICI therapy. A tailored therapeutic approach, incorporating CSs, csDMARDs, biologics and JAK inhibitors, seeks to mitigate adverse effects while preserving ICI benefits (**Figure 2**). Future advances in oncology are expected to define irAE management by elucidating irAE-arthritis mechanisms. Collaborative registries and clinical trials aim to optimize therapies that mitigate symptoms without compromising anti-tumor benefits. The continuation of ICI therapy should be individualized based on patient health, cancer progression, and arthritis response.
